# Differences in *Helicobacter pylori* and CagA antibody changes after eradication between subjects developing and not developing gastric cancer

**DOI:** 10.3164/jcbn.19-30

**Published:** 2019-06-11

**Authors:** Masaaki Kodama, Tadayoshi Okimoto, Kazuhiro Mizukami, Kensuke Fukuda, Ryo Ogawa, Kazuhisa Okamoto, Osamu Matsunari, Yoshinari Kawahara, Yuka Hirashita, Kazunari Murakami

**Affiliations:** 1 Department of Gastroenterology, Faculty of Medicine, Oita University, 1-1 Idaigaoka, Hasama-machi, Yufu, Oita 879-5593, Japan; 2 Faculty of Welfare and Health Science, Oita University, 700 Dannoharu, Oita 870-1192, Japan

**Keywords:** *Helicobacter pylori*, eradication, gastric cancer, *Helicobacter pylori* antibody, CagA antibody

## Abstract

We evaluated serological *Helicobacter pylori* and cytotoxin-associated gene A (CagA) antibodies and endoscopic atrophy after eradication to identify factors predicting post-eradication gastric cancer development. Thirty-five patients with successful eradiation were divided into the post-eradication gastric cancer (13 cases) and non-gastric cancer (22 cases) groups. Serum *Helicobacter pylori* and CagA antibody titers and endoscopic atrophy before and six years after eradication were examined. Median *Helicobacter pylori* antibody titers had decreased significantly from baseline at 0.5–2 years after eradication in both groups (gastric cancer group, from 39.0 to 11.0 U/ml, *p* = 0.011; non-gastric cancer group, from 29.6 to 4.97 U/ml, *p*<0.001), but were significantly higher in the gastric cancer than in the non-gastric cancer group (*p* = 0.029). Median serum CagA antibody titers had also decreased significantly at 0.5–2 years after eradication (gastric cancer group, from 6.35 to 3.23 U/ml, *p* = 0.028; non-gastric cancer group, from 9.88 to 1.21 U/ml, *p* = 0.0045). Serum CagA in each group showed no significance. Endoscopic atrophy improved significantly after eradication in the non-gastric cancer, but not the gastric cancer, group (*p* = 0.0007). In conclusion, changes in *Helicobacter pylori* and CagA antibody titers and endoscopic atrophy after eradication might be useful as predictive factors for post-eradication gastric cancer.

## Introduction

*Helicobacter pylori* (HP) has been proven to be a major risk factor in gastric cancer (GC).^(1,2)^ Many studies have demonstrated that HP eradication (HPE) is associated with the reduction of GC.^(3–7)^ The World Health Organization’s International Agency for Research on Cancer Working Group recognized that HP causes almost 90% of non-cardiac cancers, and that HPE decreases GC approximately 30–40% of cases.^(8)^ Thus, HPE cannot abolish the development of GC completely. Therefore, predictive and risk factors for GC discovered after HPE should be identified.

Serological evaluation with anti-HP immunoglobulin G (IgG) antibody testing is an inexpensive and noninvasive method that is used commonly throughout the world for the diagnosis of HP-related conditions.^(9)^ Because HP IgG antibody titers may remain elevated for 6–12 months after HPE, serological testing to establish HPE should be performed at least 6–12 months post-treatment.^(10)^ Serological testing has also been used in ABC risk classification to evaluate GC risk.^(11)^

Cytotoxin-associated gene A (CagA) is among the best-studied HP virulence factors.^(12)^ CagA injected into gastric epithelial cells by the bacterium binds to Src homology-2 domain-containing phosphatase and induces morphological changes and damage in these cells, which have been reported to lead to gastric carcinogenesis.^(13,14)^ Serological testing with anti-CagA antibody measurement thus provides important information about GC status.^(15)^ In one study, risk for GC development determined by HP and -CagA antibody positivity were 2.28 and 2.87 fold increases.^(15)^ Our previous study also showed that CagA seropositivity was associated closely with the risk of GC development.^(16)^

The identification of post-HPE GC risk factors would be very useful for GC screening. Several characteristics of GC arising after HPE and differences from that developing before HPE have been reported.^(17–19)^ However, almost all risk factors for post-HPE GC, including endoscopic and histological atrophy, are identified before HPE.^(20–22)^ Few reports have described GC risks detected after HPE.^(23)^ The characterization of changes in the gastric mucosa and serological status after HPE is very important for the early detection of GC development. Because serological testing is easily performed, inexpensive, and informative about GC risk, in this study we evaluated changes in serological HP and CagA antibody titers and endoscopic atrophy after HPE in the effort to identify risk factors for post-HPE GC development.

## Materials and Methods

### Subjects

We performed a retrospective, case-control study. In this study, data from patients who underwent gastrointestinal endoscopy and were diagnosed as HP positive at Oita University Hospital between January 1988 and December 2009 were evaluated. The diagnosis of HP infection was based on rapid urease test (RUT), culture, and/or histological positivity. HPE treatment was administered to all patients. Serum HP and CagA antibody status and endoscopic atrophy were evaluated in these patients with successful eradication up to seven years. The patients were retrospectively divided into GC and non-GC groups according to the development of GC after HPE. Background diseases of all patients were compared in both groups. The study protocol was approved by an institutional review board of Oita University, Faculty of Medicine (987). All procedures followed were in accordance with the ethical standards of the responsible committee on human experimentation (institutional and national) and with the Helsinki Declaration of 1964 and later versions. Informed consent or substitute for it was obtained from all patients for their being included in the study.

### Serum HP and CagA antibody titer evaluation

The serum HP and CagA antibody titers of all patients were measured. Serum HP IgG antibody titers were measured by enzyme-linked immunosorbent assay (ELISA) using the E-Plate II Eiken (Eiken Chemical Co., Ltd., Tokyo, Japan). According to the manufacturer’s instructions, values exceeding 10 U/ml were considered to be positive. Serum CagA IgG antibody titers were measured using the CagA ELISA kit (Genesis Diagnostics Ltd., Cambridgeshire, UK) and the manufacturer’s positivity threshold of >6.25 U/ml.

### Endoscopic atrophy

Endoscopic examinations were performed using Olympus electroscopes (models Q-260, Q-290, HQ-290, and others; Tokyo, Japan). Endoscopic atrophy was diagnosed using the endoscopic atrophic-border scale of Kimura and Takemoto.^(24)^ This scale correlates with histological atrophy grades determined before and after HPE:^(24–26)^ 1) closed (C) type, in which the atrophic border remains on the lesser curvature of the stomach; and 2) open (O) type, in which the atrophic border extends along the anterior and posterior walls of the stomach and is not associated with the lesser curvature. The grade of atrophy was further classified into C0, C1, C2, C3 and O1, O2, O3. Each atrophy status were scored, as C0–C3 (0 to 3) and O1–O3 (4 to 6). Score 0 indicated that atrophy was absence and score 6 indicated that atrophy was most severe.

### Statistical analysis

Statistical analyses were performed using SPSS software (SPSS Statistics 22; IBM, Armonk, NY). Titer data are expressed as medians ± SDs, and endoscopic atrophy scores are expressed as medians and interquartile ranges (IQRs). The chi-squared test and Fisher’s exact test was used to compare CagA antibody seropositivity between the GC and non-GC groups. Odds ratio and 95% confidence interval (95% CI) were calculated. Student’s *t* test and Wilcoxon signed-ranks test was used to compare groups according to antibody titers and endoscopic atrophy scores (obtained before and six years after HPE). *P* values <0.05 were considered to be significant.

## Results

### Patient characteristics

The sample comprised 35 patients. HPE treatment was successful in all patients, with negative RUT, culture, and histological results. In 13 cases (9 males, 4 females; mean age at HPE, 62.7 ± 10.4 years), GC was discovered after HPE. In 22 cases (16 males, 6 females; mean age at HPE, 55.4 ± 9.6 years), no GC developed after HPE (Table 1). The age at the time of HPE was significantly greater in the GC group than in the non-GC group (*p* = 0.021). The mean interval between HPE and the discovery of GC was 63.3 ± 56.2 months. Background upper gastrointestinal diseases (GC group/non-GC group) were chronic gastritis (5/11), gastric ulcer (GU; 4/5), duodenal ulcer (DU; 0/3), gastroduodenal ulcer (0/1), GC (3/1), gastric adenoma (1/0), and mucosa-associated lymphoid tissue lymphoma (1/0; Table 1). The GU/DU ratio did not differ between the GC (4/0) and non-GC (5/3) groups.

### Serum CagA and HP antibody seropositivity and titers

Before HPE, the rates of serum CagA antibody positivity were 61.5% (8/13) in the GC group and 54.5% (12/22) in the non-GC group, with no significant difference between groups (Table 2). The OR for CagA antibody positivity in the GC group was 1.33 (95% CI, 0.340–5.173; *p* = 0.69).

**Fig. 1:**
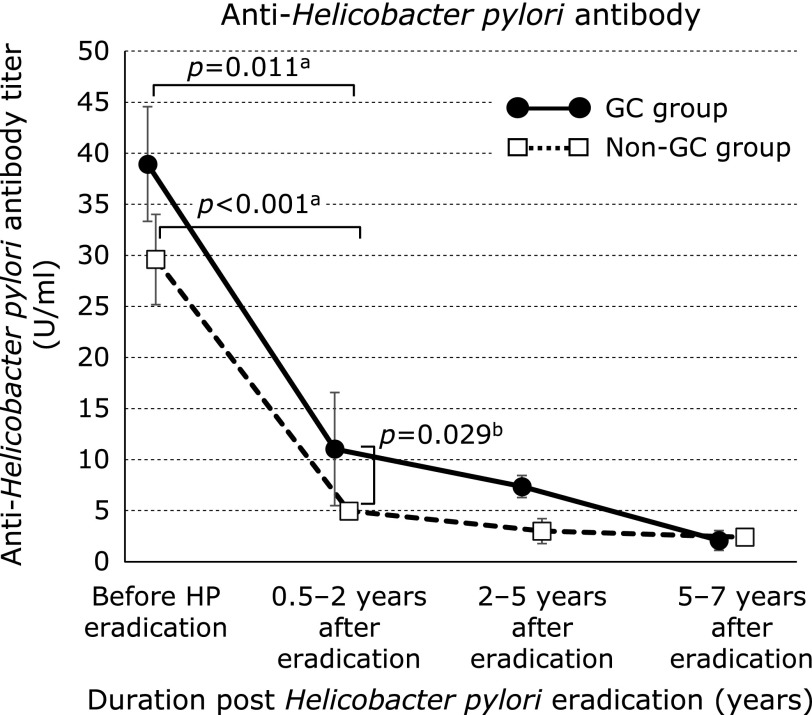
Serum *Helicobacter pylori* antibody titers in the gastric cancer and non–gastric cancer groups. Data are medians ± SDs. ^a^Wilcoxon signed-ranks test, ^b^Student’s *t* test. GC, gastric cancer.

The median serum HP antibody titers (GC group/non-GC group) were 39.0/29.6 U/ml before HPE (*p* = 0.22), 11.0/4.97 U/ml at 0.5–2 years after HPE (*p* = 0.029), 7.37/3.0 U/ml at 2–5 years after HPE (*p* = 0.26), and 2.10/2.41 U/ml at 5–7 years after HPE (*p* = 0.16; Fig. 1). Relative to pre-HPE values, HP antibody titers had decreased significantly from 0.5–2 years after HPE in both groups (GC group, *p* = 0.011; non-GC group, *p*<0.001).

**Fig. 2:**
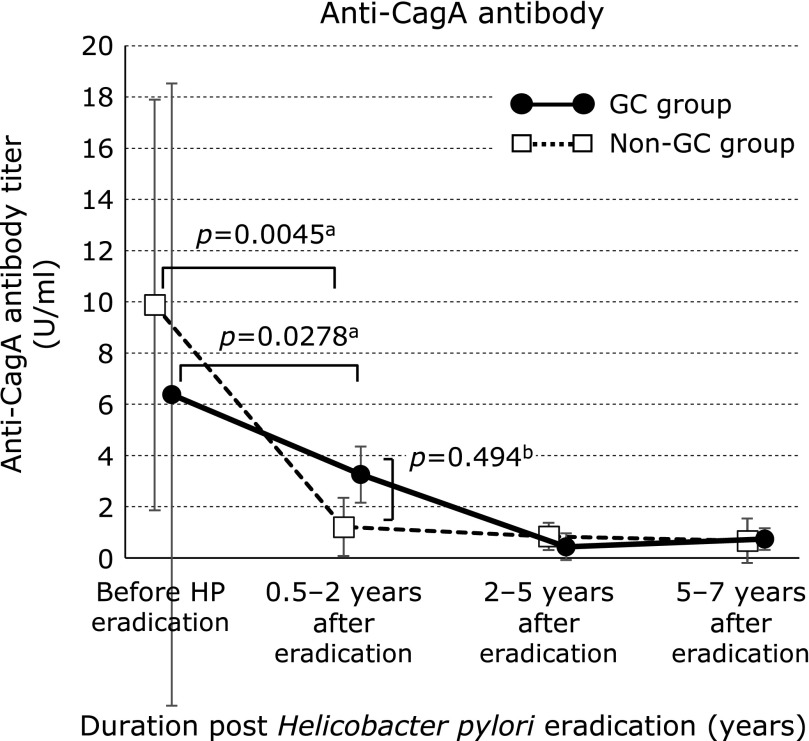
Serum CagA antibody titers in the gastric cancer and non-gastric cancer groups. Data are medians ± SDs. ^a^Wilcoxon signed-ranks test, ^b^Student’s *t* test. GC, gastric cancer.

The median serum CagA antibody titers (GC group/non-GC group) were 6.35/9.88 U/ml before HPE (*p* = 0.43), 3.23/1.21 U/ml at 0.5–2 years after HPE (*p* = 0.49), 0.42/0.83 U/ml at 2–5 years after HPE (*p* = 0.20), and 0.72/0.66 U/ml at 5–7 years after HPE (*p* = 0.34; Fig. 2). Both groups showed no significant difference during the observation period. Relative to pre-HPE values, CagA antibody titers after eradication had decreased significantly from 0.5–2 years to 5–7 years after HPE in both groups (GC group, *p* = 0.0278; non-GC group, *p* = 0.0045).

### Endoscopic atrophy

**Fig. 3:**
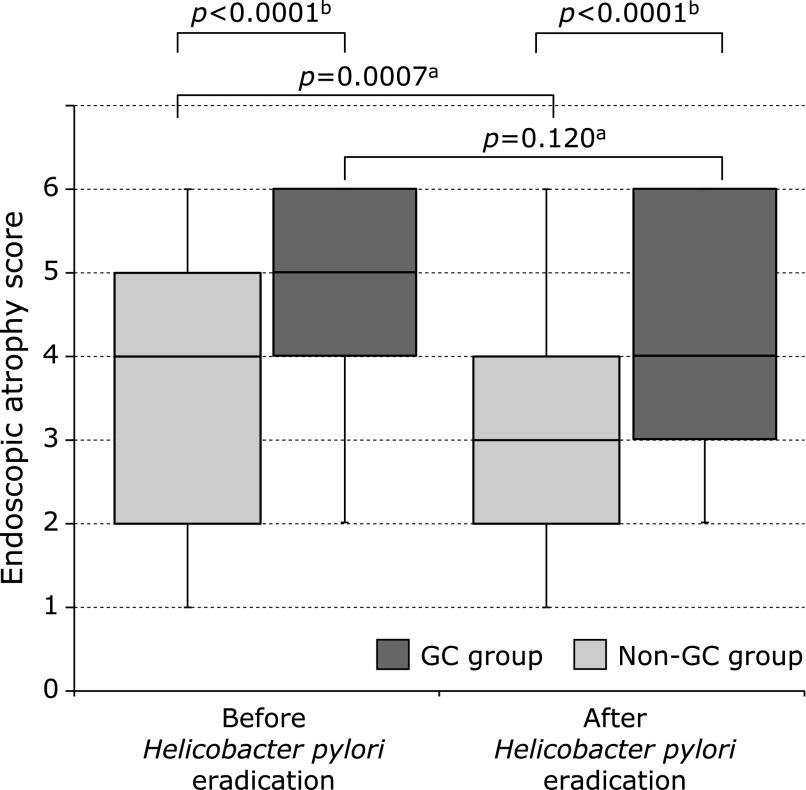
Endoscopic atrophy score before and after *Helicobacter pylori* eradication in the gastric cancer and non-gastric cancer groups. Data are medians with interquartile ranges. ^a^Wilcoxon signed-ranks test, ^b^Student’s *t* test. GC, gastric cancer.

Median endoscopic atrophy scores (GC group/non-GC group) were 5.0 (IQR, 4–6)/4.0 (IQR, 2–5) before HPE and 5.0 (IQR, 3–6)/3.0 (IQR, 2–4) at six years after HPE (Fig. 3). Atrophy scores were significantly high in the GC group before and six years after HPE (*p*<0.0001, respectively). The non-GC group showed a significant decrease in endoscopic atrophy after HPE relative to baseline (*p* = 0.0007); the GC group showed no such change (*p* = 0.12).

## Discussion

HP infection has close association with not only gastroduodenal diseases, but also many systemic inflammation and diseases. Although contradictory results were reported, several studies described that HPE improved serum lipid metabolism^(27)^ and the nutrition status in hemodialysis patients.^(28)^

HPE is known to reduce the potential for gastric carcinogenesis,^(3–8)^ but it cannot completely suppress GC development. The incidence of post-HPE GC has increased gradually with the number of subjects in whom HPE has been achieved. Although factors associated with post-HPE GC, such as advanced age at the time of HPE, male sex, severe atrophy, and intestinal metaplasia,^(4,20–22)^ have been reported, these factors are observed before HPE. Few data are available on predictive factors that are detected after HPE; only map-like erythema appearing on the gastric mucosa after HPE has been reported as a risk factor for GC development.^(23)^ In this study, changes in HP and CagA antibody titers and endoscopic atrophy were evaluated to identify post-HPE risk factors for GC.

The use of serum HP antibody testing is widespread, given its utility for mass screening for HP infection.^(9)^ However, it is unsuitable for the rapid evaluation of HPE, as titers decrease only 6–12 months after HPE.^(10)^ In this study, HP antibody titers had declined significantly at 0.5–2 years after HPE relative to baseline, and most values were <10 U/ml, in accord with previous findings.^(10)^ Although HP antibody titers were significantly higher in the GC group than in the non-GC group during this period, culture, histological and RUT findings were negative, suggesting that some cases in the GC group produced false-negative results for all tests. The rate of GC development is typically higher in patients with HP infection than in those in whom HPE has been achieved.^(3–6)^ Alternatively, HP antibody titers may have decreased more slowly in several cases in the GC group relative to the non-GC group.

Another possible explanation is related to the immunohistochemical detection of HP in the lamina propria, whereas it is usually present on the surface of the gastric mucosa.^(29,30)^ Ito *et al.*^(31)^ described the detection of HP in the gastric lymph nodes along the lesser curvature of the stomach in 21–37 of 46 patients with HP infection by culture, polymerase chain reaction, and immunohistochemistry. HP was detected in many macrophages and a few interdigitating dendritic cells. The authors suggested that the bacterium invaded through gastric mucosal damage and translocated to the gastric lymph nodes,^(31)^ with resulting effects on the immune system associated with chronic gastritis, which may in turn contribute to post-HPE gastric carcinogenesis. The slow reduction of HP antibody titers after HPE treatment may reflect a delay in the eradication of bacteria that have invaded the lymph nodes, and thus an increased risk of GC development.

CagA was the representative HP pathogenetic factor examined in this study. The risk of GC development has been shown to be higher (by 1.64 times) in patients infected with CagA-positive HP strains than in those infected with CagA-negative strains.^(32)^ The CagA protein is structurally diverse, divided into Western and East-Asian types.^(14,33)^ In Japan, most HP strains have the East-Asian type of CagA, which has stronger cytotoxicity and a closer association with gastric carcinogenesis than does the Western type.^(14,16,33,34)^ The East-Asian CagA strain has been associated significantly with severe atrophic gastritis, intestinal metaplasia, and GC development relative to the Western type.^(14,33,34)^ CagA antibody seropositivity is associated closely with GC in East-Asian countries, such as Japan, Korea, and China, despite differences in populations, the antigens used for CagA antibody.^(16)^ Therefore, the CagA antibody is a useful biomarker for GC especially in East Asia. However, the rate of seropositivity for this antibody in our sample was only 61.5%. In Japan, most HP strains are CagA positive; Shiota *et al.*^(16)^ reported that 96.3% of strains were positive for the *cagA* gene. However, extremely high CagA seropositivity is not often seen, even in East Asian countries. In our previous study, the CagA seropositivity ratio was 75.0%, and CagA antibody titers were associated significantly with the serum pepsinogen level, atrophic gastritis, and GC.^(35)^ Shimoyama *et al.*^(36)^ reported CagA antibody positivity rates of 60% (49/81) for GC cases and 44% for non-GC cases (36/81) in a Japanese sample. These rates are comparable to those found in the present study.

Although the CagA antibody seropositivity rate tended to be higher in the GC group than in the non-GC group in this study, this difference was not significant. Kikuchi *et al.*^(37)^ reported that both CagA antibody positivity and negativity were related to the risks of intestinal-type, diffuse-type, early, advanced, proximal, and distal GC in subjects with HP infection, despite the serum CagA positivity rate of about 60%. Consequently, our results also reflect the close relationship between CagA seropositivity and GC development.

In addition, 0.5–2 years after HPE, CagA antibody titers were higher in the GC group than in the non-GC group. Thus, these titers seemed to decrease more slowly in the GC group, but both groups showed no significance. As for the HP antibody titers, some cases may have yielded false-negative results. CagA is injected into the cytoplasm of gastric epithelial cells, and is detected even in nuclear fractions.^(38)^ Lim *et al.*^(39)^ described CagA translocation into human B lymphocytes. Thus, deep CagA translocation might be associated with the slow decrease in CagA antibody titers and with the development of GC.

Several studies have demonstrated the association between endoscopic and histological atrophy.^(23,24)^ In our previous study, histological atrophy and intestinal metaplasia were correlated even after HPE.^(26)^ In addition, in a previous study we found significantly more severe endoscopic atrophy in the post-HPE GC group than in the non-GC group.^(21,22)^ The present result does not contradict these previous findings. No report has described a correlation between endoscopic atrophy and GC after HPE. To our knowledge, this study is the first to demonstrate the lack of a significant change in endoscopic gastric atrophy after HPE in the GC group, in contrast to the non-GC group.

As atrophy becomes severe, intestinal metaplasia also becomes severe. Similarly, intestinal metaplasia has been reported to increase the risk of GC after HPE.^(21–23,40)^ As an improvement in intestinal metaplasia is difficult to achieve after HPE, the same may have been true for endoscopic atrophy in the GC group compared with the non-GC group. In addition, because the high HP antibody titers at 0.5–2 years after HPE suggests the presence of false-negative cases, HP and CagA localized outside the gastric mucosa might have influenced chronic inflammation in these patients.

One limitation of this study is the small sample. Further examination of larger numbers of subjects is needed to clarify the association between HP and CagA seropositivity and GC development after HPE. Second, there were few measurement opportunities during the observation period. If observation time was more frequent, the comparison of both groups may be possible.

In conclusion, significantly high HP antibody titers were observed in the short term after HPE in the GC group. Additionally, endoscopic atrophy was significantly more severe before HPE and showed no significant improvement after HPE in the GC group compared with the non-GC group. Changes in HP and CagA antibody titers and endoscopic atrophy after HPE might be useful as predictive factors for post-HPE GC development. Additional studies are needed to further clarify these associations.

## Author Contributions

MK: study concept and design, acquisition of data, analysis and interpretation of data and statistical analysis; TO, KM, and KF: acquisition of data, analysis and interpretation of data; RO, KO, OM, YK, and YH: acquisition of data; KM: drafting of the manuscript, obtained funding, administrative, technical, or material support and study supervision.

## Figures and Tables

**Table 1:** Characteristics of patients who did and did not develop gastric cancer following *Helicobacter pylori* eradication

Characteristic	Gastric cancer group	Non-gastric cancer group	*p*
Number of patients	13	22	
Sex (male/female)	9/4	16/6	0.825^a^
Age at *Helicobacter pylori* eradication (years)	62.7 ± 10.4	55.4 ± 9.6	0.021^b^
Age at gastric cancer detection (years)	67.8 ± 10.1	—	
Observation period after eradication (months)	63.3 ± 56.2	—	
Background disease at eradication			
Chronic gastritis	5	11	
GU	4	5	
DU	0	3	
Gastroduodenal ulcer	0	1	
Gastric cancer	3	1	
Gastric adenoma	0	1	
MALT lymphoma	1	0	
GU/DU	4/0	5/3	0.157^c^

Data are presented as numbers or means ± SDs. ^a^Chi-squared test, ^b^Student’s *t* test, ^c^Fisher’s exact test. GU, gastric ulcer; DU, duodenal ulcer; MALT, mucosa-associated lymphoid tissue.

**Table 2:** CagA seropositivity before *Helicobacter pylori* eradication

Group	Serum CagA antibody		OR	95% CI	*p*
	Positive	Negative			
Gastric cancer	8 (61.5%)	5	1.33	0.340–5.173	0.686^a^
Non-gastric cancer	12 (54.5%)	10			

OR, odds ratio; 95% CI, 95% confidence interval; ^a^Chi-squared test.

## Abbreviations

CagA: cytotoxin-associated gene A
DU: duodenal ulcer
ELISA: enzyme-linked immunosorbent assay
GC: gastric cancer
GU: gastric ulcer
HP: *Helicobacter pylori*
HPE: *Helicobacter pylori *eradication
IgG: immunoglobulin G
MALT: mucosa-associated lymphoid tissue
RUT: rapid urease test
